# Same-Sex Gaze Attraction Influences Mate-Choice Copying in Humans

**DOI:** 10.1371/journal.pone.0009115

**Published:** 2010-02-09

**Authors:** Jessica L. Yorzinski, Michael L. Platt

**Affiliations:** 1 Animal Behavior Graduate Group, Department of Evolution and Ecology, University of California Davis, Davis, California, United States of America; 2 Center for Cognitive Neuroscience, Duke University, Durham, North Carolina, United States of America; 3 Department of Neurobiology, Duke University Medical Center, Durham, North Carolina, United States of America; University of Sussex, United Kingdom

## Abstract

Mate-choice copying occurs when animals rely on the mating choices of others to inform their own mating decisions. The proximate mechanisms underlying mate-choice copying remain unknown. To address this question, we tracked the gaze of men and women as they viewed a series of photographs in which a potential mate was pictured beside an opposite-sex partner; the participants then indicated their willingness to engage in a long-term relationship with each potential mate. We found that both men and women expressed more interest in engaging in a relationship with a potential mate if that mate was paired with an attractive partner. Men and women's attention to partners varied with partner attractiveness and this gaze attraction influenced their subsequent mate choices. These results highlight the prevalence of non-independent mate choice in humans and implicate social attention and reward circuitry in these decisions.

## Introduction

Animals often rely on others within their group to gain valuable information about their physical and social environment. They can then use this information to guide their own decisions [1]. For example, some animals, such as chimpanzees, decide to use tools for foraging after observing other individuals manipulating these tools successfully [2]. Other animals, such as rats and mandrills, choose which foods to consume by smelling the mouths of conspecifics [3], [4].

One of the most important decisions an animal makes is the choice of a mate, and this decision can be influenced by the decisions of others–a phenomenon known as mate-choice copying [5]–[9]. Mate-choice copying can have a strong influence on mating decisions. So strong, in fact, that it can over-ride preexisting mating preferences [5]. For example, female guppies often prefer brightly-colored males over drab males; however, the same females will switch to favoring drab males if they observe other females mating with them [6], [10]. Mate-choice copying can be beneficial if it reduces mate assessment costs and improves outcomes, especially for inexperienced individuals that copy the choices of more experienced conspecifics [5]. Theory suggests that mate-choice copying can have profound affects on the evolution of sexually-selected traits [11]–[13]. Despite the potential importance of mate-choice copying, we know little about the mechanisms guiding this behavior in any species.

Recent evidence suggests that humans, like other animals, are influenced by others in their choices of mates [14]–[16]. Because attractiveness may be a reliable indicator of genotypic quality in humans [17], we hypothesized that mate-choice copying would be influenced by the attractiveness of the individuals that were being copied. If this copying occurred, evaluative conditioning could be the underlying mechanism. Evaluative conditioning is a process by which the value of a stimulus (the mate) changes depending on whether it is paired with a positive or negative stimulus (attractive or unattractive partner) [18]. We predicted that people would express a greater willingness to engage in a relationship with a potential mate (our proxy for a mating decision) if the mate was paired with an attractive partner than if the same potential mate was paired with a less attractive partner.

We also determined whether men and women's patterns of looking were associated with their proxy mating decisions. In a gaze cascade model [19], participants spend more time looking at a stimulus that they like and this attention causes them to favor the stimulus even more. We therefore predict that participants' attention towards partners will influence their mate choice decisions.

## Methods

### Participants

Thirty men and 30 women participated in this study at Duke University. They were all white, between the ages of 18 and 30 years old (mean ± SE: 22.1±0.4), and self-reported heterosexual. Flyers and emails were used to recruit participants and they were told that they would be participating in a study that explores human attractiveness. They earned $15 for their participation. The Institutional Review Board of the University of California at Davis (#200715270-1) and Duke University (#7646) approved this study; written consent was obtained for all participants.

### Stimuli

**Figure 1:**
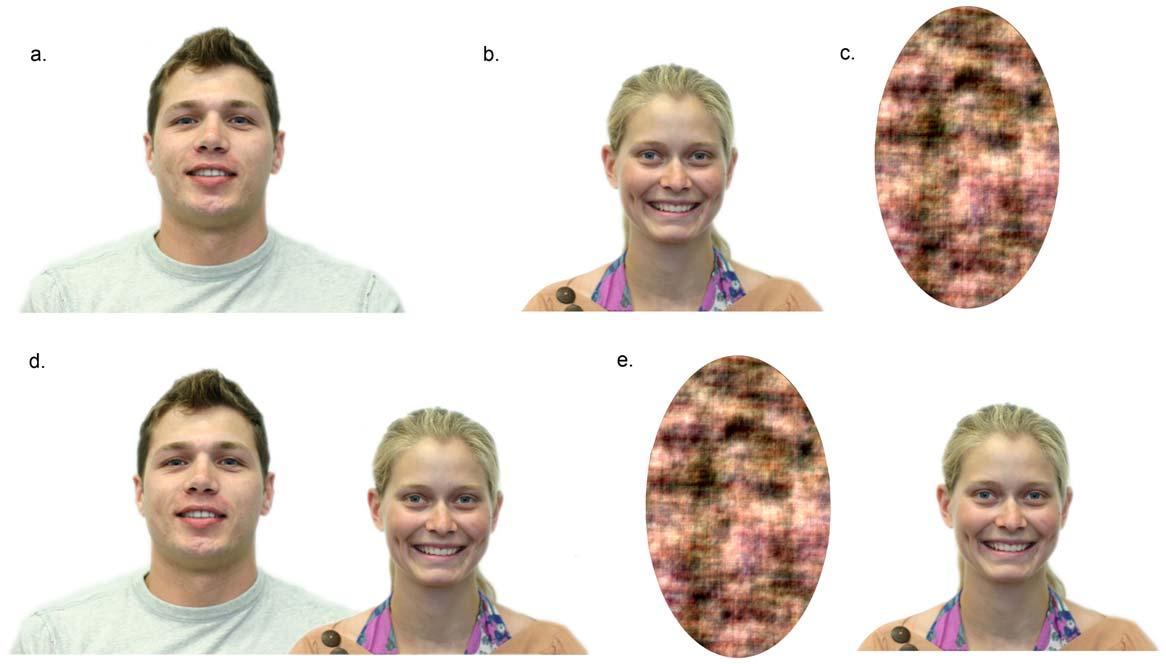
Example stimuli shown to male participants. (a), (b), and (c) were shown in part one whereas (d) and (e) were shown in part two.

Photographs of 48 men and 48 women were taken against a white background in standardized lighting conditions at the University of California at Davis (Canon EOS Digital Rebel 300D). These men and women were all white and between the ages of 18 and 30 years old (mean ± SE: 21.5±0.5). They directly faced the camera and were all smiling (with their teeth showing). The photographs were edited in Adobe Photoshop so that the men were all taller than the women (the distance between the women's chin and the bottom of the photograph was 5.4 cm and the distance between the men's chin and the bottom of the photograph was 7.2 cm; Figure 1). Thirty-six images of men and 36 images of women were created (72 images total; Figure 1a&b). An additional 12 images of men and 12 images of women were created (using a custom Matlab script) in which the people in these photographs were scrambled (the phase spectrum of the images was randomized) and the resulting scrambled image was used to color an empty oval shape (Figure 1c).

The people in these 72 images (excluding the scrambled images) were then rated for attractiveness by 35 heterosexual undergraduates (25 women and 15 men) between the ages of 18 and 30 years old (mean ± SE: 19.8±0.4) that participated in exchange for class credit. They were asked to indicate on a scale from 1 (least attractive) to 10 (most attractive) the attractiveness of the person in the photograph. They saw each photograph for five seconds and had three seconds to write-down the rating. If they recognized an individual in the photograph, their rating for that individual was omitted.

The mean attractiveness of each person in the photographs was calculated. Based on these means, the people in the photographs were assigned to one of three attractiveness categories (low attractiveness: 2.3–3.8, medium attractiveness: 3.9–4.7, and high attractiveness: 4.8–6.1) to generate 12 photographs that fell within each category for both sexes (e.g., 12 photographs displayed men that were categorized as least attractive and 12 photographs displayed women that were categorized as least attractive).

Images of men and women from the above photographs were then paired together to create a compound stimuli (male + female; the attractiveness ratings that participants assigned to the photographs of individual men and women were only used to create compound stimuli and were not used in subsequent statistical analyses). For each compound stimulus, the images of the man and woman were combined using Adobe Photoshop; the woman was positioned in front of the man (this was done to create natural-looking images of couples), and the distance between the man and woman was the same in each photograph (9 cm between the edge of the face of the woman and the adjacent edge of the face of the man; Figure 1d). The side (left or right) of the compound stimuli where the men were displayed was randomized. Each man was randomly paired in three different images with a woman that was categorized as having low attractiveness, medium attractiveness, and high attractiveness. This generated 108 compound stimuli (36 men × 3 women of each attractiveness category) in which each man and each woman were paired with someone of all three different attractiveness categories. Another 36 images were created in which each man was paired with a scrambled image of a woman and another 36 images in which each woman was paired with a scrambled image of a man (also referred to as compound stimuli; Figure 1e). All of the photographs were displayed in color.

### Experimental Design and Procedure

The study consisted of two parts and was conducted by a single researcher (JLY). In both parts, a photograph was displayed for five seconds and then the participant had three seconds to indicate a score. The first part lasted approximately 15 minutes and had two blocks. One block consisted of the 36 photographs of men that were pictured alone and the other block consisted of the 36 photographs of women that were pictured alone. In addition, the block that contained photographs which were of the same sex as the participant also had 12 photographs that displayed the scrambled images of that sex. Participants were asked to rate the attractiveness of the people in the photographs (1 = least attractive to 10 = most attractive) in the block that contained images of the same sex as the participant. They were then asked to indicate their willingness to engage in a long-term romantic relationship with the people in the photographs (1 = least willingness to 10 = most willing) in the block that contained images that were of the opposite sex as the participant. They were instructed to use the full range of the scale. They were also told to look at the scrambled images but did not have to rate them. The two blocks were displayed in a randomized order and the order of the photographs within the blocks was randomized for each participant.

In the second part of the study (approximately 40 min), the same participants saw the compound stimuli (the images in which the people were paired together). The participants were told that “the people in each photograph were engaged in a long-term romantic relationship but their relationship ended.” The participants were asked to indicate their willingness to engage in a long-term romantic relationship with the opposite-sex individual in the compound stimuli (‘mate’). The same-sex individual in the compound stimuli will be referred to as the ‘partner.’ This part was divided into 4 blocks, each of which contained 36 compound stimuli. Within each block, a given mate or partner was only displayed one time. For example, block one, two, three, and four contained a picture of a given mate with a partner of low attractiveness, medium attractiveness, high attractiveness, and a scrambled image, respectively (this ensured that each mate was paired with three different partners and one scrambled image). These four images were randomly assigned to the blocks. The blocks were randomly ordered and the order of the compound stimuli within the blocks was randomized for each participant. Participants were given a break after two blocks. In sum, a given mate was displayed five different times during each experiment (one time during part one and four times during part two); a given partner was displayed four different times during each experiment (one time during part one and three times during part two). Each participant was asked to indicate their willingness to engage in a long-term romantic relationship with 144 potential mates in part two (36 mates per block × 4 blocks) for a total of 8,640 ratings (144 mates × 60 participants).

The participants rested their hand on a track ball during the experiment. When they were prompted to indicate the score that they assigned to a person in each photograph, a screen appeared that displayed the numbers one through ten. The participants would indicate their scores by clicking on the appropriate number. The experimenter was monitoring the experiment from an adjacent room which had monitors that mirrored what the participant saw. When the participant clicked on a number, the experimenter recorded this information.

### Eye-Tracking

A ViewPoint EyeTracker® (PC-60; Arrington Research, Inc., Scottsdale, Arizona) was used to record the eye movements (right eye) of the participants during the experiment (temporal resolution: 60 Hz; dark pupil). The participants were told that we were measuring the size of their pupil but were not told that their eye movements were being monitored until after they completed the experiment. The stimuli were displayed using Viewpoint software (2.8.4) running on a Dell desktop (Precision 530 model WHL) on a 1680×1050 pixel monitor (Acer AL2216W) that was positioned 44 cm from the participant's eye. A slip correction was conducted after every photograph. The movement of the participants was minimized by having them use a bite bar (UHCOTech HeadSpot with BiteBuddy) and wrapping a Velcro strap around the back of their head that kept them in contact with the forehead rest. The equipment was calibrated (25 points) each time that the participants were positioned in front of the computer (before part one, before part two, and before the third block of part two). The resulting file consisted of coordinates of where participants were known to be looking during each sampling point.

Using a customized Matlab program, polygon regions of interest (ROI) were drawn on each photograph that outlined the entire shape of the partner and the mate. For each coordinate in the resulting file, we determined which ROI it fell within to determine whether the participant was looking at the partner, the mate, or neither of them. Because the resolution of the eye-tracking equipment was 0.5–1.0 visual degrees, the eye-tracking system was able to accurately categorize each coordinate as being within one of these three ROIs. Most importantly, we could easily resolve whether a subject was looking at the man or woman in each compound stimulus because the edges of their faces were 13.0 visual degrees apart (the degrees were calculated using 44 cm as the distance between the participant's eye and the computer screen, and using 9 cm as the distance between the people in the compound stimuli).

### Statistical Analyses

We used repeated-measures mixed model ANOVAs to evaluate mate-choice copying using PROC MIXED in SAS© 9.1. The denominator degrees of freedom was computed with the DDFM  =  BETWITHIN option in SAS, a method which divides the residual degrees of freedom into between-subject and within-subject portions; this method is recommended for large data sets with unbalanced data. In all models, ‘partner attractiveness’ was categorized as low (scores 1–3), medium (4–6), and high (7–10). We determined whether the willingness of participants to engage in a long-term romantic relationship with the mate was influenced by the mate's partner. The dependent variable was the difference between the score that participants assigned to the mate in part one and in part two (‘change in mate score’)(positive values indicate that the participant became more willing to engage in a long-term romantic relationship with the mate after seeing the mate beside the partner). The independent variables were the sex of the participant, the attractiveness scores that participants assigned to the partner in part one (‘partner attractiveness’), the interaction between these two variables (‘sex of participant’ * ‘partner attractiveness’), and the score that participants assigned to the mate in part one (‘initial mate score’). Because the same mate was displayed four times (with different partners) to each participant in part two, the mate was included as a random variable that was nested within participant (this was done in all subsequent models to account for repeated measures). Least-squares means (LSMEANS) were calculated to examine sex differences in the change in mate's score with respect to the attractiveness of the partner (a Bonferonni correction was performed when multiple comparisons were made); in particular, we determined the change in mate score when partner attractiveness was low and high.

We examined the relationship between the eye-tracking results and partner attractiveness. In each compound stimulus, only two people were displayed on a white background in each photograph. Not surprisingly, subjects spent most of their time (89.7±0.001%) looking at one person or the other. Therefore, the amount of time spent looking at a partner (‘looking at partner’) was calculated as the total amount of time spent looking at the partner divided by the sum of the amount of time participants spent looking at both the partner and mate. ‘Gaze shift’ was the number of times participants looked back and forth between the partner and mate. We ran two mixed models for men and women with ‘looking at partner’ and ‘gaze shifts’ as the dependent variables; the independent variables were ‘partner attractiveness’ and ‘initial mate score.’

Lastly, we assessed the relationship between all three variables: ‘gaze shift’/‘looking at partner’, ‘partner attractiveness,’ and ‘change in mate score.’ Because men and women evaluated partners differently (see Results), the dependent variable was ‘gaze shift’ for men and ‘time looking at partner’ for women. The independent variable was the interaction between the ‘change in mate score’ and ‘partner attractiveness’ as well as ‘initial mate score.’

The mate choice scores and eye-tracking data were also analyzed with respect to the control (scrambled) images. The models assessed whether participants treated the controls differently than they treated the partners. Graphs display means and standard errors.

## Results

### Women Show Stronger Mate-Choice Copying than Men

**Figure 2:**
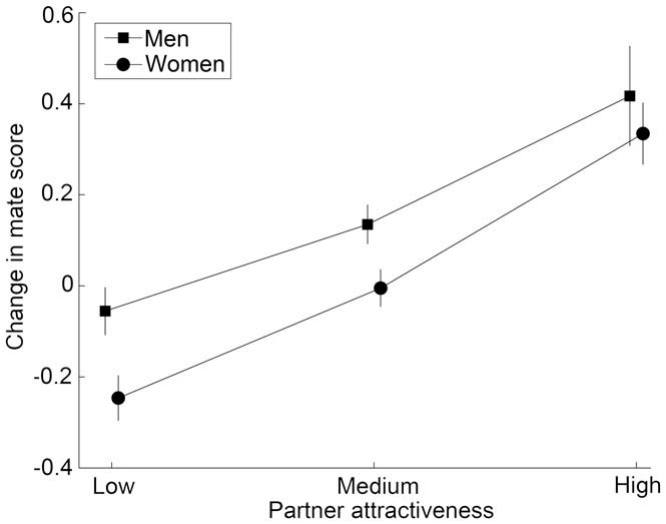
Mate-choice copying in women and men depends on partner attractiveness (means ± SE).

Both female and male participants showed mate-choice copying. Specifically, the willingness of a participant to engage in a long-term romantic relationship with a mate (our proxy of a mating decision) varied with the attractiveness of the mate's partner. Participants were more willing to engage in a long-term romantic relationship with a mate when the mate was paired with an attractive partner (F_2,109_ = 85.68, p<0.0001; n = 60 (30 women 30 men); Figure 2). This change in the willingness of participants to engage in a long-term romantic relationship with a mate varied with the initial mate score (F_1,6357_ = 395.46, p<0.0001) and tended to vary with the sex of the participant (F_1,58_ = 3.47, p = 0.07).

The change in mate score varied significantly with the interaction between sex of the participant and attractiveness of the mate's partner (F_2,109_ = 17.31, p<0.0001). When the attractiveness of the partner was low, women were less willing to engage in long-term romantic relationship with a mate (t(109) = 6.18, adjusted p-value<0.0001); men did not give different scores to a mate when the mate was displayed alone and when the mate was paired with an unattractive partner (t(109) = 0.08, adjusted p-value = 1.0). When the attractiveness of the partner was high, men and women were more willing to engage in a long-term romantic relationship with a mate (men: t(109) = 4.04, adjusted p-value<0.0001; women: t(109) = 6.86, adjusted p-value<0.0001).

Even though a given mate was displayed multiple times with different partners, the overall results were unaffected. They were qualitatively the same when the order in which each mate was displayed in each of the four blocks during part two (first, second, third, or fourth) was included as another independent variable. Because people can have multiple long-term relationships (either sequentially or concurrently) [20], the experimental design was consistent with human relationship patterns. Participants were equally likely to engage in a long-term romantic relationship with a mate when the mate was displayed beside a control stimulus compared to when it was displayed alone (t(59) = 1.92, p = 0.06).

### Gaze Patterns Reflect Attractiveness

**Figure 3:**
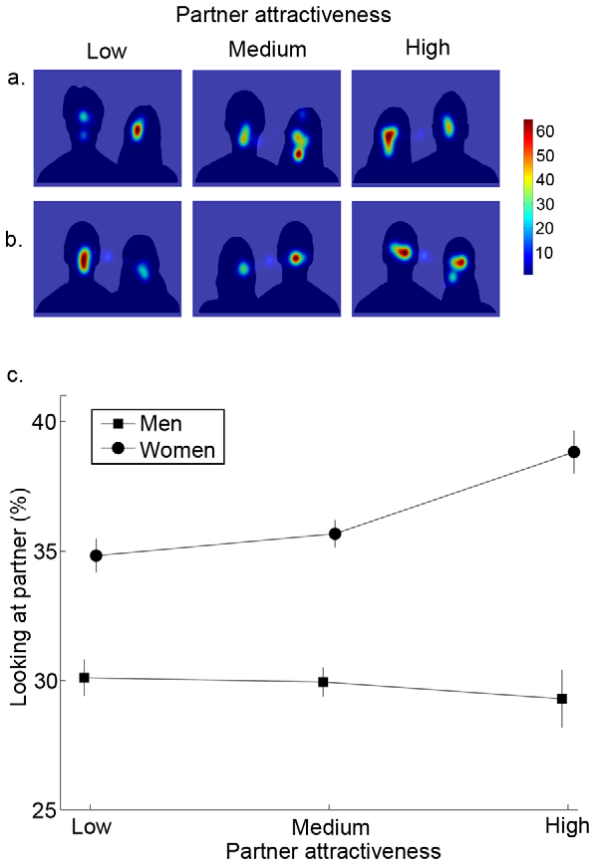
Gaze patterns of men and women with respect to partner attractiveness. In (a) and (b), example heat maps indicate where men and women, respectively, look when viewing the compound stimuli as a function of low, medium, and high partner attractiveness (the taller figures in each compound stimuli represent the men). Men spend similar amounts of time looking at partners irrespective of partner attractiveness but women spend more time looking at partners that are highly attractive (c)(means ± SE).

**Figure 4:**
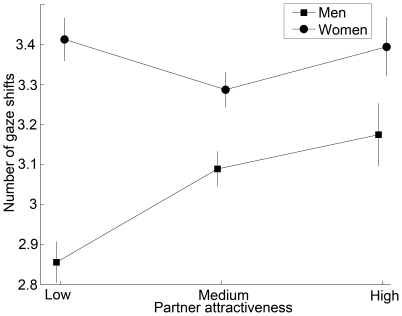
For men, frequency of shifting gaze between mate and partner depends on partner attractiveness (means ± SE).

Overall, both male and female participants spent more time looking at a mate compared to the partner (t(59) = 20.10, p<0.0001). Nonetheless, attention to the partner varied with attractiveness. Specifically, women spent more time looking at the partner when the partner attractiveness was higher (F_2,55_ = 8.9, p = 0.0004) while men spent similar amounts of time looking at the partner regardless of the partner attractiveness (F_2,54_ = 0.11, p = 0.90; Figure 3). In contrast, men more often shifted their gaze between the partner and mate when partner attractiveness was high (F_2,54_ = 5.82, p = 0.0051); however, women's frequency of gaze shifts did not relate to partner attractiveness (F_2,55_ = 1.23, p = 0.30; Figure 4).

As a control, we examined patterns of gaze when potential mates were paired with an oval-shaped control stimulus (see Methods). Both male and female participants spent more time looking at partners compared to control stimuli (F_1,59_ = 2298, p<0.0001) and shifted their gaze between a mate and control stimulus less often than they did between a mate and partner (F_1,59_ = 2872, p<0.0001), thus indicating that attention to a partner depended on its quality as a social stimulus with identifiable human features.

### Gaze Patterns Predict Mate Choice Decisions

**Figure 5:**
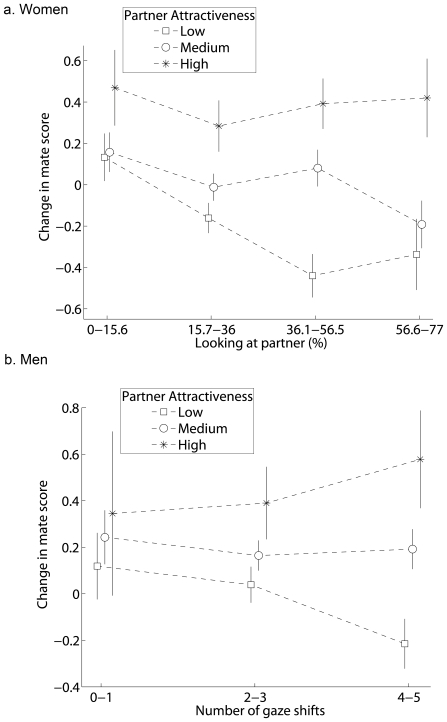
Women and men were more likely to increase their score of the mate when they directed less attention to partners with low attractiveness (means ± SE). For graphical purposes only, the amount of time looking at the partner was pooled into groups containing −2 SE, −1 SE, +1 SE, and +2 SE from the mean (36%) and the number of gaze shifts was pooled into groups of two.

The gaze patterns of men and women predicted their mate choice decisions. Because men and women evaluated partners differently (see above), we determined whether either frequency of gaze shifts (for men) or the amount of time spent looking at partners (for women) varied with mate choice and partner attractiveness. Gaze patterns were influenced by the interaction between partner attractiveness and change in mate score (men: F_3,2867_ = 6.48, p = 0.0002; women: F_3,2981_ = 4.43, p = 0.0041). When partner attractiveness was low, both men and women were more likely to increase their score of the mate when they directed less attention to the partner (women spent less time looking at the partner: (t(2981) = 2.98, adjusted p-value = 0.0058); men shifted their gaze between the partner and mate less often: (t(2867) = 3.43, adjusted p-value = 0.00012)). When partner attractiveness was high, men and women's attention to the partner did not predict their mate choice decision (men: t(2867) = 1.30, adjusted p-value = 0.38; women: t(2981) = 0.42, adjusted p-value = 1.0; Figure 5).

## Discussion

We found that proxy mating decisions made by people were strongly influenced by the attractiveness of partners depicted with potential mates. Specifically, men and women were more likely to express interest in a long-term relationship with a potential mate when that mate was paired with an attractive partner. These results are consistent with other studies of mate-choice copying in humans. Jones et al. [14] found that women rated men in photographs as being more attractive when a woman was smiling at that man (the converse was found for male participants). Regardless of the initial attractiveness of the men, Waynforth [15] found that women were more likely to find men attractive when those men were pictured with attractive women compared to when they were pictured with unattractive women. Lastly, Little et al. [16] showed that both male and female participants found a mate more desirable in a long-term relationship when the mate was shown beside attractive partners. Our study is different from these previous studies in that we investigated both men and women's mate-choice copying behavior while considering their initial mate-choice preferences and monitoring their gaze.

We found that men and women differed slightly in their mate-choice copying behavior. Women showed an overall greater reliance on the decisions of same-sex partners than did men, although both were influenced by partner attractiveness. This pattern was especially prominent when the attractiveness of the same-sex partner was low: women were less interested in engaging in a long-term relationship with the mate while men's interest in the mate was not different from their initial evaluations. Because females are generally more selective in their choice of mates compared to men (due to differential parental investment) [21] they may be more skeptical of mates paired with unattractive partners while males may have a high baseline interest in all potential mates.

In addition, men and women differed in their gaze patterns while they evaluated the same-sex partners. The amount of time spent looking at partners influenced the women's mating decisions but the number of times looking back and forth between the partner and mate affected the men's mating decisions. These gaze differences could reflect differences between men and women in processing visual social information. Because men can process information about attractiveness faster than women [22], they may be able to gather information about same-sex partners with brief gaze shifts. Alternatively, shifting gaze could reflect men's vigilance, which may vary with the presence of a partner and his attractiveness, and thus index intrasexual competition. Cross-cultural studies could indicate whether mate-choice copying is widespread across cultures, even in societies where women are unable to freely choose their mates.

Overall, our results also align with previous studies on mate-choice copying in non-human animals. Females of species from diverse taxonomic groups change their mating decisions based upon the mating choices of other females [23]. The mating decisions of males can also be influenced by conspecific males [24]. However, these studies primarily investigate whether the mere presence of another same-sex conspecific affects an individual' mate choice decision. In contrast, studies on humans, including our own, have shown that humans do not just prefer mates that are chosen by others but consider the attractiveness of the individuals that they are copying. Because humans often have sexual relationships with more than one person [25], extracting specific information (such as attractiveness) about that person's previous partner is likely more useful than merely assessing whether a previous partner existed. The same may be true in other pair-bonding species.

**Figure 6:**
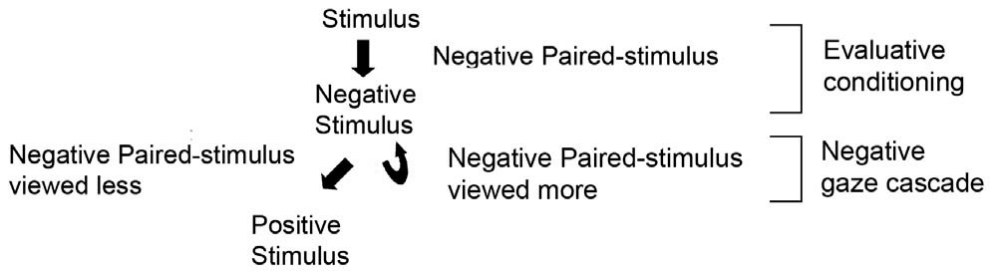
Schematic model based on evaluative conditioning and a gaze cascade that explains mate-choice copying and attention. A stimulus that is paired with a negative stimulus has a negative value (evaluative conditioning). A negative paired-stimulus continues to have a negative effect on the stimulus when it is viewed more frequently (negative gaze cascade) compared to when it is viewed less frequently.

Participants' proxy mating decisions can be incorporated with our gaze results to suggest that evaluative conditioning along with a modified gaze cascade model partially drives mate-choice copying in humans. Our data support an evaluative conditioning mechanism: a mate (stimulus) paired with an unattractive partner (negative paired-stimulus) became less valuable (the willingness of participants to engage in a relationship with the mate decreased) whereas a mate paired with an attractive partner (positive paired-stimulus) became more valuable (the willingness of participants to engage in a relationship with the mate increased). This phenomenon is similar to techniques used in consumer marketing: advertisements can be more successful when a product (stimulus) is associated with a highly attractive woman (positive paired-stimulus) [26]. Furthermore, our gaze data suggest a modified version of the gaze cascade model [19] that incorporates this evaluative conditioning. When a mate (stimulus) was paired with an unattractive partner (negative paired-stimulus), the unattractive partner continued to have a negative influence on the mate when participants directed their attention toward it; however, the unattractive partner did not have a negative influence on the mate when the participants directed less attention toward it (Figure 6). This cascade model does not fit with positive paired-stimuli (attention directed toward attractive partners did not influence the value of the mate), possibly indicating that attractiveness had already reached an asymptotic level.

Although we are unaware of any neurobiological study of mate-choice copying, the above results along with the prevalence of the phenomenon across different taxa suggest the involvement of brain regions that encode social reward information and contribute to attention. It has already been demonstrated that one such region, the medial orbitofrontal cortex, is activated when people view attractive faces of both sexes. This activation is even stronger when the faces display positive expressions [27]. The nucleus accumbens is also activated when men view attractive women [28]. Moreover, neurons in the parietal cortex that mediate attention encode the value of orienting to social and reproductive stimuli, enhancing the likelihood of looking at salient social cues [29].

These observations suggest the interaction of brain areas involved in social reward and attention during mate-choice copying. Brain regions that compute reward value may be engaged when evaluating a mate and signals may be further enhanced depending on partner attractiveness and visual attention. Such signals presumably inform processing in brain areas, such as parietal cortex, that guide attention [29]. Overtly orienting to social stimuli would then further modulate processing in reward-related areas of the brain, in a straightforward neurobehavioral feedback loop. Future neurobiological studies will be necessary to evaluate this model.
